# Electroacupuncture at different frequencies (5Hz and 25Hz) ameliorates cerebral ischemia-reperfusion injury in rats: possible involvement of p38 MAPK-mediated anti-apoptotic signaling pathways

**DOI:** 10.1186/s12906-015-0752-y

**Published:** 2015-07-18

**Authors:** Chin-Yi Cheng, Jaung-Geng Lin, Nou-Ying Tang, Shung-Te Kao, Ching-Liang Hsieh

**Affiliations:** School of Chinese Medicine, College of Chinese Medicine, China Medical University, Taichung, 40402 Taiwan; Department of Chinese Medicine, Hui-Sheng Hospital, 42056 Taichung, Taiwan; Department of Chinese Medicine, China Medical University Hospital, 40447 Taichung, Taiwan; Graduate Institute of Integrated Medicine, College of Chinese Medicine, China Medical University, 91 Hsueh-Shih Road, Taichung, 40402 Taiwan; Research Center for Chinese Medicine & Acupuncture, China Medical University, Taichung, 40402 Taiwan

**Keywords:** Electroacupuncture, Mitogen-activated protein kinases, Phospho-p38 MAPK, Bcl-2, Bcl-xL, X-linked inhibitor of apoptosis protein

## Abstract

**Background:**

This study aimed to determine the effects of electroacupuncture stimulation at the Baihui (GV20) and Fengfu (GV16) acupoints, at frequencies of 5Hz (EA-5Hz) and 25Hz (EA-25Hz), 7 days after cerebral ischemia-reperfusion (I/R) injury, and to evaluate the possible signaling mechanisms involved in mitogen-activated protein kinase (MAPK) pathways.

**Methods:**

Rats were subjected to 30 min of middle cerebral artery occlusion (MCAo) followed by 7 days of reperfusion. EA-5Hz or EA-25Hz was applied immediately after MCAo and then once daily for 7 consecutive days.

**Results:**

Results indicated that EA-5Hz and EA-25Hz both markedly attenuated cerebral infarction and neurological deficits. EA-5Hz and EA-25Hz both markedly downregulated cytosolic glial fibrillary acidic protein (GFAP), mitochondrial Bax, mitochondrial and cytosolic second mitochondrial-derived activator of caspase/direct inhibitor of apoptosis protein-binding protein with low isoelectric point (Smac/DIABLO), and cytosolic cleaved caspase-3 expression, and effectively restored cytosolic phospho-p38 MAPK (p-p38 MAPK), cytosolic cAMP response element-binding protein (CREB), mitochondrial Bcl-xL, and cytosolic X-linked inhibitor of apoptosis protein (XIAP) expression, in the ischemic cortical penumbra 7 days after reperfusion. Both EA-5Hz and EA-25Hz also significantly increased the ratios of mitochondrial Bcl-xL/Bax and Bcl-2/Bax, respectively.

**Conclusions:**

Both EA-5Hz and EA-25Hz effectively downregulate reactive astrocytosis to provide neuroprotection against cerebral infarction, most likely by activating the p38 MAPK/CREB signaling pathway. The modulating effects of EA-5Hz and EA-25Hz on Bax-mediated apoptosis are possibly due to the activation of p38 MAPK/CREB/Bcl-xL and p38 MAPK/CREB/Bcl-2 signaling pathways, respectively, and eventually contribute to the prevention of Smac/DIABLO translocation and subsequent restoration of XIAP-mediated suppression of caspase-3 in the cortical periinfarct area 7 days after reperfusion.

## Background

Apoptosis in the penumbra, occurring through various signaling pathways, is considered the major cause of cerebral infarct expansion following ischemia-reperfusion (I/R) injury [1, 2]. Mitogen-activated protein kinases (MAPKs), including c-Jun N-terminal kinase (JNK), extracellular signal-regulated kinase1/2 (ERK1/2), and p38 MAPK, are mediators of various cellular signaling pathways in response to extracellular apoptotic stimuli and intracellular oxidative stress after transient focal cerebral ischemia [3, 4]. MAPKs might play roles in the regulation of cell death and survival, depending on the cell type and cerebral ischemic model [5]. Studies have reported that p38 MAPK activation initiates glutamate-mediated neurotoxicity after focal cerebral ischemia, and that selective inhibition of p38 MAPK signaling cascades provides neuroprotection against cerebral I/R injury [3, 6]. Studies have also indicated that p38 MAPK activation upregulates the expression of the cAMP response element-binding protein (CREB)-regulated cell survival proteins Bcl-2 and Bcl-xL in cerebral ischemic preconditioning models [7–9]. The Bcl-2 family proteins, including Bcl-2, Bcl-xL, and Bax, play pivotal roles in regulating mitochondria-mediated apoptotic pathway. Neuronal death or survival is determined by the balance between proapoptotic (Bax) and antiapoptotic (Bcl-2 and Bcl-xL) proteins during cerebral ischemia [10, 11]. Studies have suggested that Bax heterodimerization with Bcl-2 and Bcl-xL is essential for antiapoptotic activity in the central nervous system [11]. Following activation of the apoptotic signaling cascade after cerebral I/R injury, Bax/Bcl-2 and Bax/Bcl-xL heterodimers are rapidly dissociated, and the monomeric form of Bax translocates from the cytosol to the mitochondrial membrane, where it is cross-linked as a Bax homodimer, and opens the mitochondrial permeability transition pore. Pore opening leads to the release of cytochrome c and second mitochondrial-derived activator of caspase (Smac)/direct inhibitor of apoptosis -binding protein with low isoelectric point (DIABLO) from the mitochondrial intermembrane space to the cytosol [10, 12]. The release of cytochrome c and dATP-dependent formation of apoptotic protease activating factor-1 (Apaf-1)/caspase-9 complex (apoptosome) then initiates the activation of downstream effector caspases [13]. The inhibitor of apoptosis protein (IAP) family members, including X-linked IAP (XIAP), c-IAP1, c-IAP2, and Survivin, participate in apoptosis regulation by directly binding to caspases (−3, −7, and −9). Of all IAP family members, XIAP has the most potent antiapoptotic properties [14]. During cerebral ischemia, mature Smac/DIABLO interacts with XIAP in the cytosol, which abrogates the anticaspase function of XIAP and triggers caspase-3 dependent apoptosis [15].

Electroacupuncture (EA) combines traditional acupuncture and modern electrotherapy, and is effective at treating various ailments, including stroke [16]. Studies have shown that EA stimulation at acupoints at various frequencies exerts neuroprotective effects against cerebral I/R injury by activating ERK1/2 [17–19] or p38 MAPK signaling [20]. According to traditional Chinese medicine theory, the Baihui (GV20) and Fengfu (GV16) acupoints both lie on the Governing Vessel, which connects directly to the brain. These acupoints are commonly used to treat brain disorders. In Lu et al. (2010), EA stimulation at Fengfu and Fengchi (GB20) acupoints, at a frequency of 2/15Hz, provided neuroprotective effects by downregulating S100β-mediated neurotoxicity during craniocerebral tumor resection [21]. In studies on experimental stroke rats, EA stimulation at the Baihui acupoint (2/15Hz) reduced glutamate toxicity [22] and exerted antiapoptotic effects by increasing the Bcl-2/Bax ratio [16] during the acute phase, and EA stimulation (3Hz) improved behavioral performance by increasing brain-derived neurotrophic factor (BDNF) production [23] during the subacute phase after middle cerebral artery occlusion (MCAo). Tian et al. (2013) demonstrated that EA stimulation at the Baihui, Mingmen (GV4), and Zusanli (S36) acupoints (30/50Hz) provided neuroprotection against brain edema through the activation of Na^+^, K^+^-ATPase in rats after transient global cerebral ischemia [24].

Therefore, in this study, we aimed to evaluate the effects of EA stimulation at the Baihui and Fengfu acupoints (EA at acupoints), at a frequency of 5 or 25Hz, after 30 min of cerebral ischemia followed by 7 days of reperfusion, and to evaluate the possible involvement of MAPK cascades in the ischemic cortical penumbra.

## Methods

### Experimental animals

Male Sprague Dawley (SD) rats weighing 300–350 g (aged approximately 8–9 weeks) were used in experiments. They were maintained at a humidity of 55 % ± 5 % on a 12 h light–dark cycle, at 22 ± 2 °C. All experimental procedures were performed in accordance with the ethical guidelines approved by the China Medical University Institutional Animal Care and Use Committee (Permit Number: 102-249-c), and the committee recognized that the study design and proposed experimental procedures followed the Animal Protection Law by the Council of Agriculture, Executive Yuan, Taiwan. All the experimental procedures involving animals avoided or minimized discomfort, pain, and stress in animals.

### Transient middle cerebral artery occlusion

The MCAo model was established in the SD rats by using intraluminal suture methods as described previously [25]. Briefly, the rats were anesthetized with a 5 % isoflurane-oxygen mixture, maintained on a 2 % isoflurane-oxygen mixture, and the right distal middle cerebral artery (MCA) was exposed through a cranial burr hole (2.5 mm lateral and 2.0 mm posterior to the bregma). After dissection, the right common carotid artery (CCA) and internal carotid artery (ICA) were exposed, and the pterygopalatine artery was ligated close to its origin. A 3–0 nylon thread with a blunt tip, made by heating near a flame, was inserted into the ICA to block blood flow to the MCA. After 30 min of MCAo, the suture was carefully removed to restore blood flow. Blood flow in the MCA was monitored by Laser-Doppler flowmetry (DRT4, Moor Instruments Inc, Wilmington, USA) through the cranial burr hole in the preischemia (>500 units), ischemia (<100 units), and reperfusion (>300 units) periods. These data were used to confirm the success of the cerebral I/R procedure.

### Electrode implantation

Before the MCAo operation, the head of each anesthetized rat was fixed to a stereotactic frame. The electrodes consisted of two stainless steel wires (diameter 0.5 mm) used for acupoint (or nonacupoint) stimulation. Electrodes were implanted in the Baihui (midpoint of the parietal bone, 4 mm depth, forward insertion) and Fengfu (the depression below the spinous process of the second cervical vertebra, 7.5 mm depth, vertical insertion) acupoints [26], or in bilateral costal regions (nonacupoints).

### Evaluation of neurological function

The neurological score of each rat was evaluated after 1, 3, and 7 days of reperfusion. Motor, sensory, balance, and reflex functions were evaluated using modified neurological severity scores, as described previously [27]. The overall neurological function of each rat was graded using a numerical scale from 0 to 18 (reference score, 0; maximal deficit score, 18).

### Experiment A

#### Grouping

The rats were randomly divided into Sham, Model, EA-5Hz, Non-acup-5Hz, EA-25Hz, and Non-acup-25Hz groups (*n* = 5). The rats in the EA-5Hz group were subjected to MCAo and simultaneously received EA at acupoints at a frequency of 5 Hz (EA-5Hz). The rats were received the first EA at acupoints for 25 min during MCAo. They were subjected to 30 min of ischemia followed by reperfusion. After 1 day of reperfusion, the rats received EA-5Hz (25 min) once daily for 6 (total 7) consecutive days, and were sacrificed 7 days after reperfusion. The rats in the Non-acup-5Hz group were subjected to the same procedures as the rats in the EA-5Hz group but received EA at nonacupoints (Non-acup-5Hz). The rats in the EA-25Hz group were subjected to the same procedures as the rats in the EA-5Hz group but received EA at acupoint at a frequency of 25Hz (EA-25Hz). The rats in the Non-acup-25Hz group were subjected to the same procedures as the rats in the EA-25Hz group but received EA at nonacupoints (Non-acup-25Hz). The rats in the Model group were subjected to the same procedures as the rats in the EA-5Hz group but did not receive EA. The rats in the Sham group were subjected to the same procedures as the rats in the Model group but the MCA origin was not occluded.

#### Electroacupuncture at acupoints or nonacupoints

An electrical stimulator (Trio 300, ITO Co, Germany) was used to generate EA at acupoints (EA-5Hz and EA-25Hz) or nonacupoints (Non-acup-5Hz and Non-acup-25Hz) for 25 min once daily for 7 consecutive days. The stimulation parameters were a 5- or 25-Hz amplitude-modulated wave of 2.7-3.0 mA intensity and 150-μs pulse width. During EA at acupoints or nonacupoints, the rats were fully awake in the cages.

#### Assessment of cerebral infarction

Following neurological assessment 1, 3, and 7 days after reperfusion, the rats were sacrificed. Their brains were removed immediately and cut into 6 coronal 2-mm sections. The sections were stained with 2 % 2,3,5-triphenyltetrazolium chloride (TTC; Merck, Germany) for 5 min at 37 °C. The brain tissues were differentiated according to white infarct and red noninfarct areas, and the cerebral infarct areas were measured using image analysis software (ImageJ, Java). The ratio of cerebral infarction areas/total brain areas was then calculated.

### Experiment B

#### Grouping

The rats were randomly divided into 6 groups: Sham, Model, EA-5Hz, Non-acup-5Hz, EA-25Hz and Non-acup-25Hz groups (*n* = 4). They were then subjected to the experimental procedures described in Experiment A.

#### Western blot analysis

Seven days after reperfusion, the rats were sacrificed, and their brains were removed and then coronally sectioned from −4.3 to +1.7 mm bregma. The right ischemic cortex was carefully separated into its penumbra (frontoparietal cortex chosen between 3 mm and 8 mm to the ischemic core) and ischemic core fractions. The right penumbral cortex was further separated into cytosolic and mitochondrial fractions according to the manufacturer’s instructions (#K256-100 BioVision, USA). The protein concentrations in the cytosolic and mitochondrial fractions were determined using the Bio-Rad assay. The samples were subjected to gel electrophoresis and transferred to a nitrocellulose membrane in western blot analysis as described previously [28]. The transferred membranes were then incubated with a rabbit anti-SAPK/JNK (JNK; 1:1000 dilution, #9252 Cell Signaling Technology), rabbit anti-phospho-SAPK/JNK (p-JNK (Thr183/Tyr185); 1:1000 dilution, #9251 Cell Signaling Technology), rabbit anti-p44/42 MAPK (ERK; 1:1000 dilution, #9102 Cell Signaling Technology), rabbit anti-phospho-p44/42 MAPK (p-ERK; 1:1000 dilution, #9101 Cell Signaling Technology), rabbit anti-p38 MAP kinase (p38 MAPK; 1:1000 dilution, #9212 Cell Signaling Technology), rabbit anti-phospho-p38 MAPK (p-p38 MAPK (Thr180/Tyr182); 1:1000 dilution, #9211 Cell Signaling Technology), rabbit anti-Akt (1:1000 dilution, #4685 Cell Signaling Technology), rabbit anti-phospho-Akt (p-Akt (Ser473); 1:1000 dilution, #9271 Cell Signaling Technology), rabbit anti-heat shock protein 70 (HSP70; 1:1000 dilution, #4872 Cell Signaling Technology), mouse anti-glial fibrillary acidic protein (GFAP; 1:1000 dilution, #3670 Cell Signaling Technology), rabbit anti-CREB (1:1000 dilution, #9197 Cell Signaling Technology), mouse anti-phospho-CREB (p-CREB; 1:500 dilution, DAM1482729 Millipore), mouse anti-p53 (1:500 dilution, ab26 Abcam), rabbit anti-Bcl-2 (1:1000 dilution, #2876 Cell Signaling Technology), rabbit anti-Bax (1:1000 dilution, #2772 Signaling Technology), rabbit anti-Bcl-xL (1:1000 dilution, #2762 Cell Signaling Technology), rabbit anti-Smac/DIABLO (1:1000 dilution, ab8114 Abcam), mouse anti-cytochrome c (1:200 dilution, 257–100 BioVision), rabbit anti-cleaved caspase-8 (1:1000 dilution, 3259–100 BioVision), rabbit anti-XIAP (1:1000 dilution, #2042 Cell Signaling Technology), rabbit anti-cleaved caspase-3 (1:1000 dilution, #9661S Cell Signaling Technology), or rabbit anti-apoptosis-inducing factor (AIF; 1;1000 dilution, #4642 Cell Signaling Technology) antibody overnight at 4 °C. The transferred membranes were also probed with antibodies specific for mouse anti-actin (1:5000 dilution, MAB1501 Chemicon) and mouse anti-cytochrome c oxidase subunit IV (COX IV; 1:5000 dilution, AB14744-100 Abcam), as an internal control for the cytosolic and mitochondrial fractions, respectively. After washing, the membranes were incubated with either an anti-rabbit horseradish peroxidase (HRP)-conjugated IgG (1:5000 dilution, Jackson ImmunoResearch) or an anti-mouse HRP-conjugated IgG (1:5000 dilution, Santa Cruz Biotechnology) antibody for 1 h at room temperature (RT). Blots were then developed with an enhanced chemiluminescence reagent (ECL-plus GE Healthcare) on a luminescence image analyzer (LAS-3000, FujiFilm). Densitometric analysis was performed using ImageJ analysis software. Results were expressed quantitatively as optical density ratios of proteins/actin or COX IV.

#### Immunofluorescent (IF) costaining

After 30 min of cerebral ischemia followed by 7 days of reperfusion, the rats were sacrificed under deep anesthesia. After transcardial perfusion with 4 % paraformalaldehyde (pH 7.4), the rat brains were removed immediately. The procedure for preparing brain sections was performed as described previously [29]. Brain sections were blocked using 10 % normal animal serum (ScyTek, Logan, Utah, USA) in Dulbecco’s phosphate buffered saline (DPBS; Sigma-Aldrich) for 20 min at RT. They were then incubated with a mouse anti-phospho-CREB (p-CREB; 1:200 dilution, DAM1482729 Millipore) antibody overnight at 4 °C. After washing 3 times with DPBS, the sections were incubated with a DyLight 594-conjugated AffiniPure goat anti-mouse IgG antibody (red, 1:400 dilution, Jackson ImmunoResearch) for 1 h at RT. The p-CREB-stained sections were counterstained with 4',6-diamidino-2-phenylindole (DAPI; 1:1000 dilution, Sigma-Aldrich, USA, nuclear staining) for 10 min at RT. Finally, all sections were mounted in an aqueous mounting medium (Aquatex, HC886685 Merck) and the cortical periinfarct areas were viewed under a fluorescent microscope (CKX41, Olympus). Sections incubated without the p-CREB primary antibodies were analyzed as the negative controls.

#### Statistical analysis

Data are expressed as mean ± standard deviation (SD). All variables were approximately normally distributed and all parametric testing, such as analysis of variance (ANOVA), was appropriate. Data from all experimental groups were compared using a one-way ANOVA followed by post-hoc analysis by using the Scheffe test. A *P* value of less than 0.05 indicates a statistically significant difference.

## Results

### Effects of EA-5Hz and EA-25Hz on cerebral infarct area

**Fig. 1:**
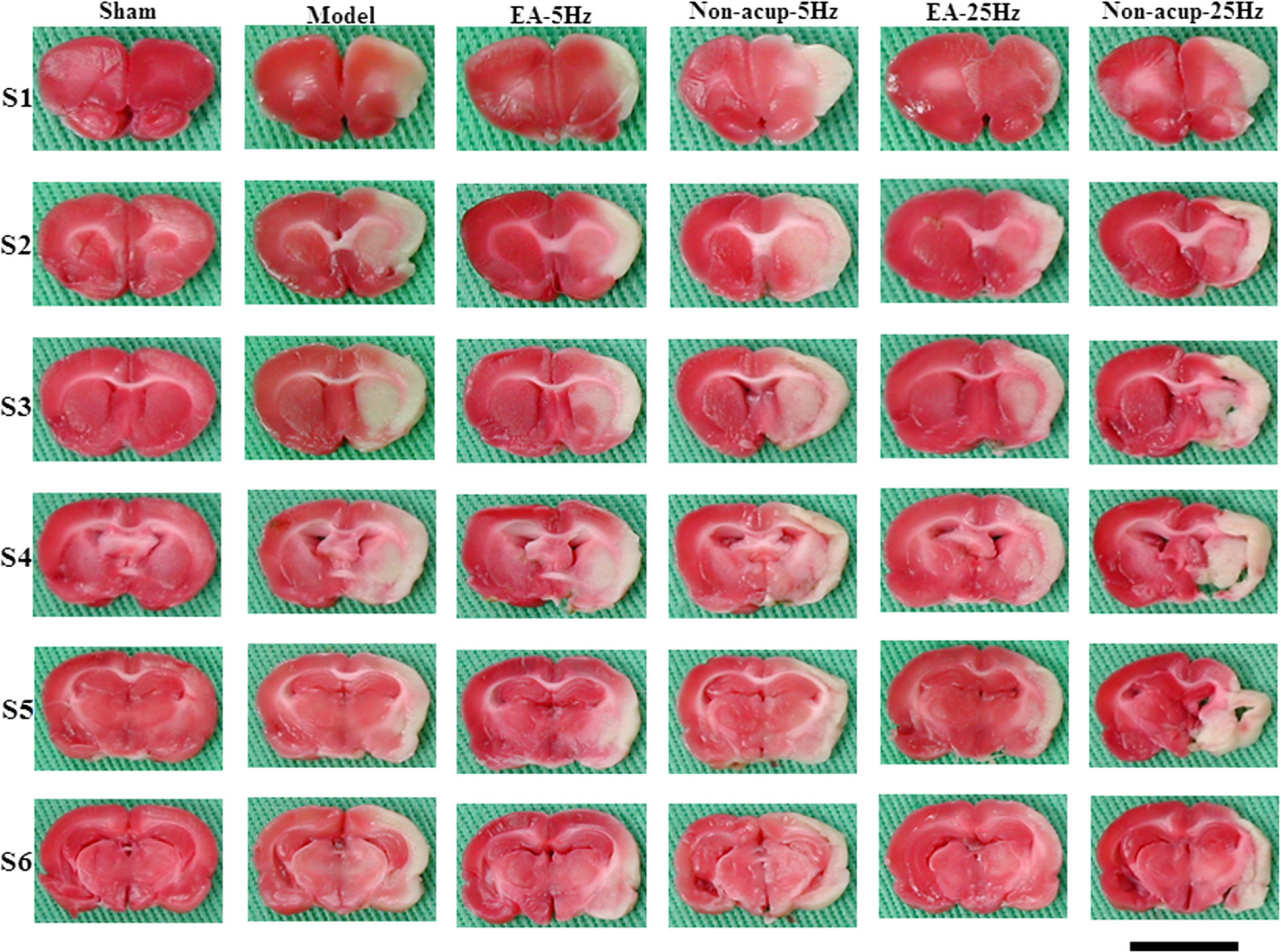
Focal cerebral infarct areas (S1-S6) in the experimental groups after 30 min of ischemia followed by 7 days of reperfusion. 2,3,5-Triphenyltetrazolium chloride staining shows noninfarct (red) and infarct (white) regions. Scale bar = 1 cm

**Fig. 2:**
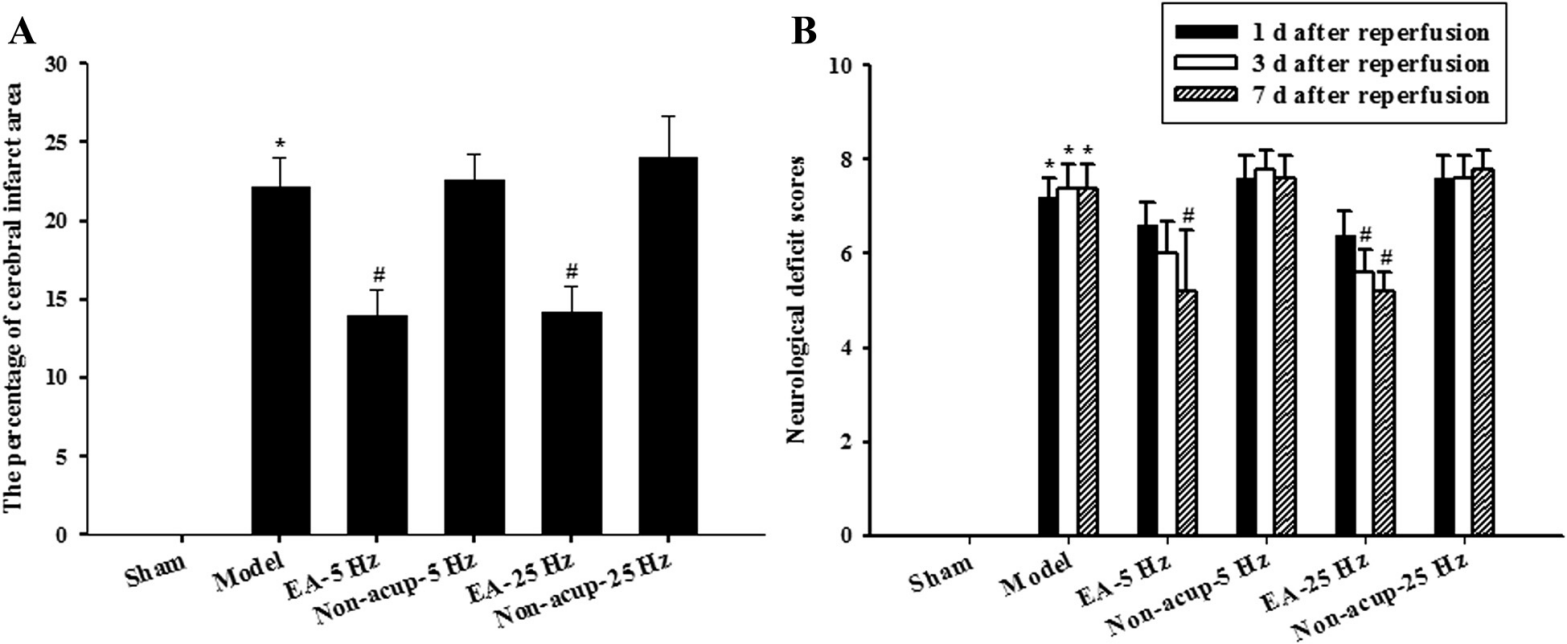
Effects of EA-5Hz and EA-25Hz on cerebral infarction and neurological function 7 days after reperfusion. **a** The percentage cerebral infarct areas in the Sham, Model, EA-5Hz, Non-acup-5Hz, EA-25Hz, and Non-acup-25Hz groups were assessed 7 days after reperfusion (*n* = 5). **b** The neurological deficit scores in the Sham, Model, EA-5Hz, Non-acup-5Hz, EA-25Hz, and Non-acup-25Hz groups were evaluated 1, 3, and 7 days after reperfusion. Data are presented as mean ± SD. *P < 0.05 compared with the Sham group; #*P* < 0.05 compared with the Model group

The rats developed prominent cerebral infarction after 30 min of MCAo followed by 7 days of reperfusion (*P* < 0.05 vs. Sham group; Figs. 1 and 2a). The percentage cerebral infarct areas were significantly lower in the EA-5Hz and EA-25Hz groups than in the Model group 7 days after reperfusion (both *P* < 0.05; Figs. 1 and 2a). However, the percentage cerebral infarct areas in the Model, Non-acup-5Hz, and Non-acup-25Hz groups exhibited nonsignificant differences (*P* > 0.05).

### Effects of EA-5Hz and EA-25Hz on neurological function

The rats had moderate neurological impairment after 30 min of MCAo followed by 1 day of reperfusion, as indicated by neurological deficit scores of approximately 6.4-7.6. The neurological deficit scores of the Model, EA-5Hz, Non-acup-5Hz, EA-25Hz, and Non-acup-25Hz groups exhibited nonsignificant differences (*P* > 0.05; Fig. 2b). After 3 days of reperfusion, the neurological deficit scores of the EA-25Hz group were significantly lower than those of the Model group (*P* < 0.05; Fig. 2b). The neurological deficit scores of the Model, Non-acup-5Hz and Non-acup-25Hz groups exhibited nonsignificant differences (*P* > 0.05). After 7 days of reperfusion, the neurological deficit scores of the EA-5Hz and EA-25Hz groups were significantly lower than those of the Model group (both *P* < 0.05; Fig. 2b). However, the neurological deficit scores of the Model, Non-acup-5Hz and Non-acup-25Hz groups showed nonsignificant differences (*P* > 0.05).

### Effects of EA-5Hz and EA-25Hz on the cytosolic expression of phosphorylated MAPKs, nonphosphorylated MAPKs, p-Akt, and Akt

**Fig. 3:**
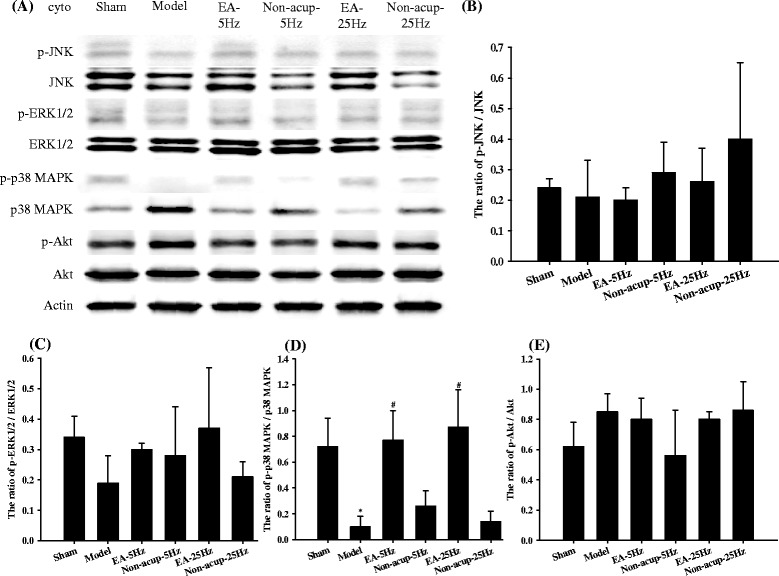
Effects of EA-5Hz and EA-25Hz on the cytosolic expression of p-JNK, JNK, p-ERK1/2, ERK1/2, p-p38 MAPK, p38 MAPK, p-Akt, and Akt in the ischemic cortical penumbra. **a** Representative western blot images show the cytosolic expression of p-JNK, JNK, p-ERK1/2, ERk1/2, p-p38 MAPK, p38 MAPK, p-Akt, and Akt in the ischemic cortical penumbra in the Sham, Model, EA-5Hz, Non-acup-5Hz, EA-25Hz, and Non-acup-25Hz groups 7 days after reperfusion. Actin was used as an internal control. The relative cytosolic expression of **b** p-JNK/JNK, **c** p-ERK1/2/ERK1/2, **d** p-p38 MAPK/p38 MAPK, and **e** p-Akt/Akt was measured in the ischemic cortical penumbra in the Sham, Model, EA-5Hz, Non-acup-5Hz, EA-25Hz, and Non-acup-25Hz groups (*n* = 4). cyto, cytosolic fraction. Data are presented as mean ± SD. *P < 0.05 compared with the Sham group; #*P* < 0.05 compared with the Model group

Western blot analysis of the ischemic cortical penumbra revealed nonsignificant differences in the ratios of cytosolic p-JNK/JNK, p-ERK1/2/ERK1/2, and p-Akt/Akt among the experimental groups 7 days after reperfusion (*P* > 0.05; Figs. 3a, 3b, 3c, and 3e). The ratio of cytosolic p-p38 MAPK/p38 MAPK expression was significantly lower in the Model group (0.1-fold) than in the Sham group (*P* < 0.05), and significantly higher in the EA-5Hz and EA-25Hz groups (7.7-fold and 8.7-fold, respectively) than in the Model group (both *P* < 0.05; Figs. 3a and 3d). The ratio of cytosolic p-p38 MAPK/p38 MAPK expression in the Model, Non-acup-5Hz, and Non-acup-25Hz groups exhibited nonsignificant differences (*P* > 0.05). The cytosolic expression patterns of p-p38 MAPK were opposite to those of p38 MAPK in all experimental groups (Fig. 3a).

### Effects of EA-5Hz and EA-25Hz on the cytosolic expression of HSP70, GFAP, p-CREB/CREB, and p53

**Fig. 4:**
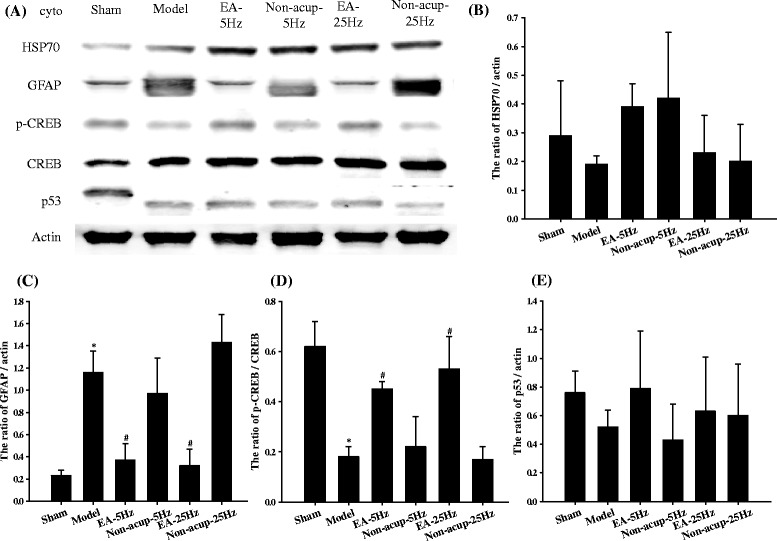
Effects of EA-5Hz and EA-25Hz on the cytosolic expression of HSP70, GFAP, p-CREB, CREB, and p53 in the ischemic cortical penumbra. **a** Representative images show the cytosolic expression of HSP70, GFAP, p-CREB, CREB, and p53 in the ischemic cortical penumbra in the Sham, Model, EA-5Hz, Non-acup-5Hz, EA-25Hz, and Non-acup-25Hz groups 7 days after reperfusion. The relative cytosolic expression of **b** HSP70, **c** GFAP, **d** p-CREB/CREB, and **e** p53 was measured in the ischemic cortical penumbra in the Sham, Model, EA-5Hz, Non-acup-5Hz, EA-25Hz, and Non-acup-25Hz groups (*n* = 4). Data are presented as mean ± SD. *P < 0.05 compared with the Sham group; #*P* < 0.05 compared with the Model group

We observed nonsignificant differences in the cytosolic expression of HSP70 and p53 in ischemic cortical penumbra 7 days after reperfusion among the experimental groups (*P* > 0.05; Figs. 4a, 4b, and 4e). Cytosolic GFAP expression was markedly higher in the Model group (5.0-fold) than in the Sham group (*P* < 0.05), and significantly lower in the EA-5Hz (0.3-fold) and EA-25Hz (0.3-fold) groups than in the Model group (both *P* < 0.05; Figs. 4a and 4c). Cytosolic GFAP expression in the Model, Non-acup-5Hz, and Non-acup-25Hz groups showed nonsignificant differences (*P* > 0.05). The ratio of cytosolic p-CREB/CREB expression was significantly lower in the Model group (0.3-fold) than in the Sham group (*P* < 0.05), and significantly higher in the EA-5Hz and EA-25Hz groups (2.5-fold and 2.9-fold, respectively) than in the Model group (both *P* < 0.05; Figs. 4a and 4d). The ratio of cytosolic p-CREB/CREB expression in the Model, Non-acup-5Hz, and Non-acup-25Hz groups exhibited nonsignificant differences (*P* > 0.05).

### Effects of EA-5Hz and EA-25Hz on the cytosolic expression of proapoptotic (Bax) and antiapoptotic (Bcl-2 and Bcl-xL) proteins

**Fig. 5:**
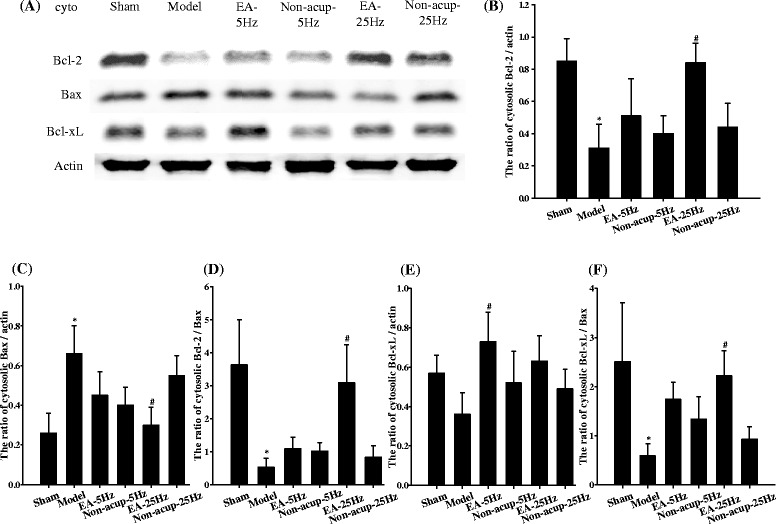
Effects of EA-5Hz and EA-25Hz on the cytosolic expression of Bcl-2, Bax, and Bcl-xL in the ischemic cortical penumbra. **a** Representative images show the cytosolic expression of Bcl-2, Bax, and Bcl-xL in the ischemic cortical penumbra in the Sham, Model, EA-5Hz, Non-acup-5Hz, EA-25Hz, and Non-acup-25Hz groups 7 days after reperfusion. The relative cytosolic expression of **b** Bcl-2, **c** Bax, **d** Bcl-2/Bax, **e** Bcl-xL, and **f** Bcl-xL/Bax was measured in the ischemic cortical penumbra in the Sham, Model, EA-5Hz, Non-acup-5Hz, EA-25Hz, and Non-acup-25Hz groups (*n* = 4). Data are presented as mean ± SD. *P < 0.05 compared with the Sham group; #*P* < 0.05 compared with the Model group

Cytosolic Bcl-2 expression in the ischemic cortical penumbra 7 days after reperfusion was significantly lower in the Model group (0.4-fold) than in the Sham group (*P* < 0.05), and significantly higher in the EA-25Hz group (2.7-fold) than in the Model group (*P* < 0.05; Figs. 5a and 5b). Cytosolic Bax expression was significantly higher in the Model group (2.5-fold) than in the Sham group (*P* < 0.05), and significantly lower in the EA-25Hz group (0.5-fold) than in the Model group (*P* < 0.05; Figs. 5a and 5c). Cytosolic Bcl-2 and Bax expression in the Model, EA-5Hz, Non-acup-5Hz, and Non-acup-25Hz groups showed nonsignificant differences (*P* > 0.05). Cytosolic Bcl-xL expression was significantly higher in the EA-5Hz group (2.0-fold) than in the Model group (*P* < 0.05; Figs. 5a and 5e). However, cytosolic Bcl-xL expression in the Model, Non-acup-5Hz, EA-25Hz, and Non-acup-25Hz groups exhibited nonsignificant differences (*P* > 0.05). The ratios of cytosolic Bcl-2/Bax and Bcl-xL/Bax expression were significantly lower in the Model group (0.1-fold and 0.2-fold, respectively) than in the Sham group (both *P* < 0.05), and significantly higher in the EA-25Hz group (5.9-fold and 3.8-fold, respectively) than in the Model group (both *P* < 0.05; Figs. 5d and 5f). The ratios of cytosolic Bcl-2/Bax and Bcl-xL/Bax expression in the Model, EA-5Hz, Non-acup-5Hz, and Non-acup-25Hz groups exhibited nonsignificant differences (*P* > 0.05).

### Effects of EA-5Hz and EA-25Hz on the mitochondrial expression of proapoptotic (Bax) and antiapoptotic (Bcl-2 and Bcl-xL) proteins

**Fig. 6:**
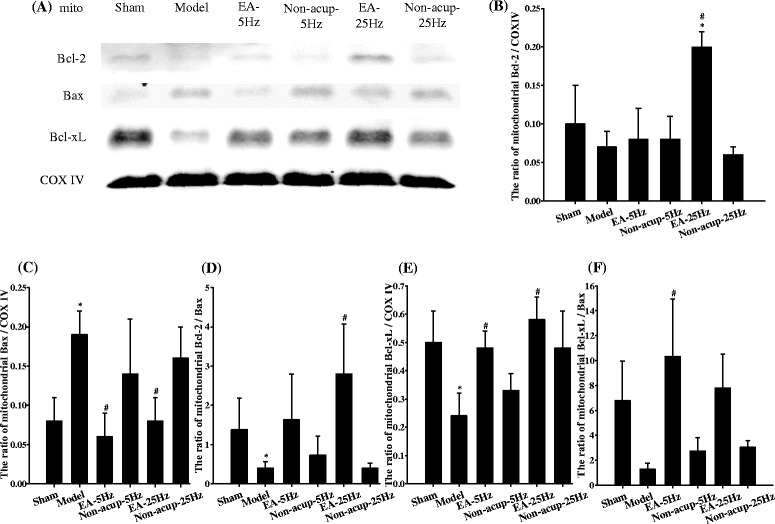
Effects of EA-5Hz and EA-25Hz on the mitochondrial expression of Bcl-2, Bax, and Bcl-xL in the ischemic cortical penumbra. **a** Representative images show the mitochondrial expression of Bcl-2, Bax, and Bcl-xL in the ischemic cortical penumbra in the Sham, Model, EA-5Hz, Non-acup-5Hz, EA-25Hz, and Non-acup-25Hz groups 7 days after reperfusion. COX IV was used as an internal control. The relative mitochondrial expression of **b** Bcl-2, **c** Bax, **d** Bcl-2/Bax, **e** Bcl-xL, and **f** Bcl-xL/Bax was measured in the ischemic cortical penumbra in the Sham, Model, EA-5Hz, Non-acup-5Hz, EA-25Hz, and Non-acup-25Hz groups (*n* = 4). mito, mitochondrial fraction. Data are presented as mean ± SD. *P < 0.05 compared with the Sham group; #*P* < 0.05 compared with the Model group

Mitochondrial Bcl-2 expression in the ischemic cortical penumbra 7 days after reperfusion was significantly higher in the EA-25Hz group than in the Sham and Model groups (2.0-fold and 2.9-fold, respectively) (both *P* < 0.05; Figs. 6a and 6b). Mitochondrial Bcl-2 expression in the Sham, Model, EA-5Hz, Non-acup-5Hz, and Non-acup-25Hz groups exhibited nonsignificant differences (*P* > 0.05). Mitochondrial Bax expression was significantly higher in the Model group (2.4-fold) than in the Sham group (*P* < 0.05), and significantly lower in the EA-5Hz (0.3-fold) and EA-25Hz (0.4-fold) groups than in the Mode group (both *P* < 0.05; Figs. 6a and 6c). By contrast, mitochondrial Bcl-xL expression was significantly lower in the Model group (0.5-fold) than in the Sham group (*P* < 0.05), and significantly higher in the EA-5Hz (2.0-fold) and EA-25Hz (2.4-fold) groups than in the Model group (both *P* < 0.05; Figs. 6a and 6e). Mitochondrial Bax and Bcl-xL expression in the Model, Non-acup-5Hz, and Non-acup-25Hz groups showed nonsignificant differences (*P* > 0.05). The ratio of mitochondrial Bcl-2/Bax expression was significantly lower in the Model group (0.3-fold) than in the Sham group (*P* < 0.05), and markedly higher in the EA-25Hz group (7.0-fold) than in the Model group (*P* < 0.05; Fig. 6d). The ratio of mitochondrial Bcl-xL/Bax was markedly higher in the EA-5Hz group (8.2-fold) than in the Model group (*P* < 0.05; Fig. 6f). Mitochondrial Bcl-2/Bax and Bcl-xL/Bax expression in the Model, Non-acup-5Hz, and Non-acup-25Hz groups exhibited nonsignificant differences (*P* > 0.05).

### Effects of EA-5Hz and EA-25Hz on the mitochondrial and cytosolic expression of Smac/DIABLO, cytochrome c, and AIF

**Fig. 7:**
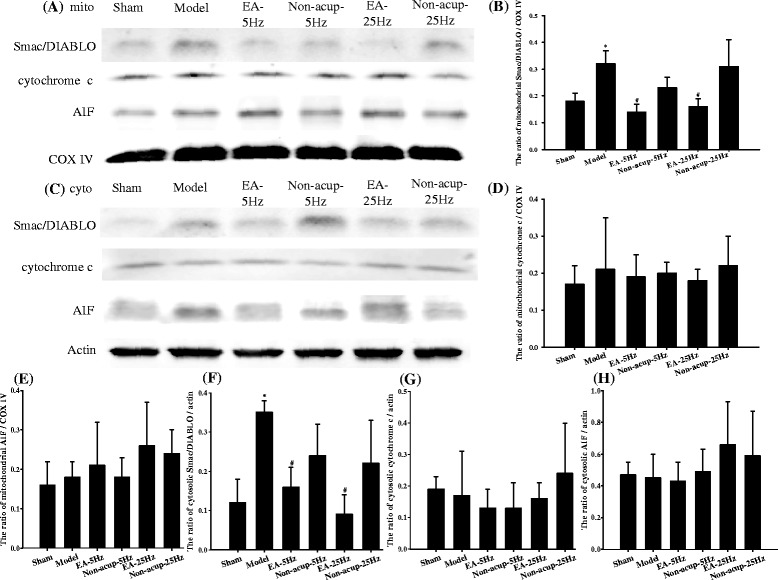
Effects of EA-5Hz and EA-25Hz on the mitochondrial and cytosolic expression of Smac/DIABLO, cytochrome c, and AIF in the ischemic cortical penumbra. Representative images show **a** mitochondrial and **c** cytosolic expression of Smac/DIABLO, cytochrome c, and AIF in the ischemic cortical penumbra in the Sham, Model, EA-5Hz, Non-acup-5Hz, EA-25Hz, and Non-acup-25Hz groups 7 days after reperfusion. The relative **b** mitochondrial Smac/DIABLO, **d** mitochondrial cytochrome c, **e** mitochondrial AIF, **f** cytosolic Smac/DIABLO, **g** cytosolic cytochrome c, and **h** cytosolic AIF expression was measured in the ischemic cortical penumbra in the Sham, Model, EA-5Hz, Non-acup-5Hz, EA-25Hz, and Non-acup-25Hz groups (*n* = 4). Data are presented as mean ± SD. *P < 0.05 compared with the Sham group; #*P* < 0.05 compared with the Model group

Mitochondrial and cytosolic Smac/DIABLO expression in ischemic cortical penumbra 7 days after reperfusion was significantly higher in the Model group (1.8-fold and 2.9-fold, respectively) than in the Sham group (both *P* < 0.05), and significantly lower in the EA-5Hz (0.4-fold and 0.5-fold, respectively) and EA-25Hz (0.5-fold and 0.3-fold, respectively) groups than in the Model group (all *P* < 0.05; Figs. 7a, 7b, 7c, and 7f). However, mitochondrial and cytosolic Smac/DIABLO expression in the Model, Non-acup-5Hz, and Non-acup-25Hz groups exhibited nonsignificant differences (*P* > 0.05). Mitochondrial and cytosolic expression of cytochrome c and AIF showed nonsignificant differences among the experimental groups (*P* > 0.05; Figs. 7a, 7c, 7d, 7e, 7g, and 7h).

### Effects of EA-5Hz and EA-25Hz on the cytosolic expression of cleaved caspase-8, XIAP, and cleaved caspase-3

**Fig. 8:**
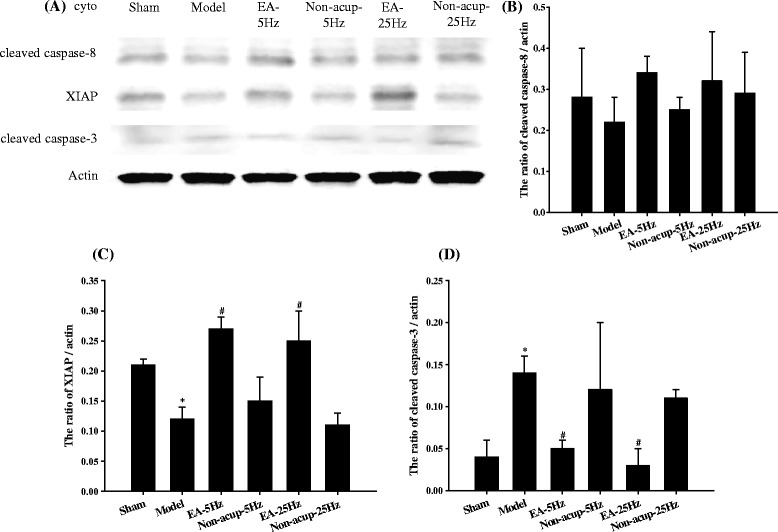
Effects of EA-5Hz and EA-25Hz on the expression of cytosolic cleaved caspase-8, XIAP, and cleaved caspase-3 in the ischemic cortical penumbra. **a** Representative images show the expression of cleaved caspase-8, XIAP, and cleaved caspase-3 in the cytosolic fraction of the ischemic cortical penumbra in the Sham, Model, EA-5Hz, Non-acup-5Hz, EA-25Hz, and Non-acup-25Hz groups 7 days after reperfusion. The relative cytosolic expression of **b** cleaved caspase-8, **c** XIAP, and **d** cleaved caspase-3 was measured in the ischemic cortical penumbra in the Sham, Model, EA-5Hz, Non-acup-5Hz, EA-25Hz, and Non-acup-25Hz groups (*n* = 4). Data are presented as mean ± SD. *P < 0.05 compared with the Sham group; #*P* < 0.05 compared with the Model group

We observed nonsignificant differences in the cytosolic expression of cleaved caspase-8 in ischemic cortical penumbra 7 days after reperfusion among the experimental groups (*P* > 0.05; Figs. 8a and 8b). Cytosolic XIAP expression was significantly lower in the Model group (0.6-fold) than in the Sham group (*P* < 0.05), and significantly higher in the EA-5Hz (2.3-fold) and EA-25Hz (2.1-fold) groups than in the Model group (both *P* < 0.05; Figs. 8a and 8c). Cytosolic cleaved caspase-3 expression was significantly higher in the Model group (3.5-fold) than in the Sham group (*P* < 0.05), and significantly lower in the EA-5Hz (0.4-fold) and EA-25Hz (0.2-fold) groups than in the Model group (both *P* < 0.05; Figs. 8a and 8d). We observed nonsignificant differences in the cytosolic expression of XIAP and cleaved caspase-3 among the Model, Non-acup-5Hz, and Non-acup-25Hz groups (*P* > 0.05). The cytosolic expression patterns of XIAP were opposite to those of cleaved caspase-3 expression in all experimental groups (Fig. 8a, 8c, and 8d).

### Expression of p-CREB/DAPI doubled-labeled cells in the ischemic cortical penumbra

**Fig. 9:**
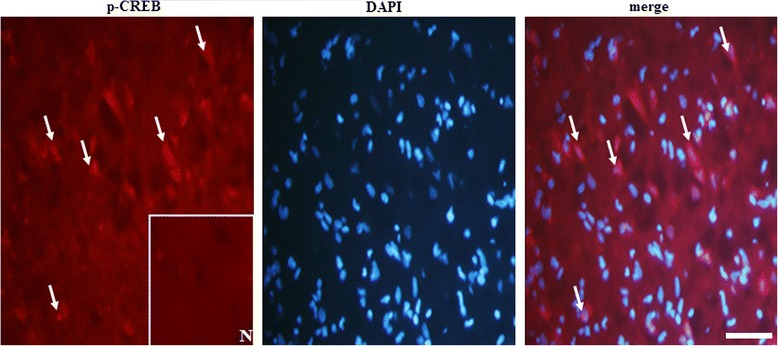
Expression of p-CREB/DAPI doubled-labeled cells in the ischemic cortical penumbra. Representative photographs show p-CREB-immunoreactive cells and DAPI-stained nuclei. The merged image shows p-CREB immunoreactivity and DAPI-stained nuclei in the ischemic cortical penumbra 7 days after reperfusion. N, negative control. The white arrows indicate intense p-CREB (red) immunoreactivity and p-CREB/DAPI staining (merged image). Scale bar = 5 μm

Seven days after reperfusion, analyses of p-CREB/DAPI IF costaining indicated strong cytoplasmic p-CREB immunoreactivity and intense nuclear p-CREB immunoreactivity in the ischemic cortical penumbra (Fig. 9).

## Discussion

In this study, we observed that 30 min of MCAo produced gross cerebral infarction in the MCA territory 7 days after reperfusion. These results are consistent with those of a previous study, which showed a large cerebral infarction caused by delayed infarct expansion in the subacute phase after mild transient focal cerebral ischemia [1]. Our results indicated that both EA-5Hz and EA-25Hz, applied immediately after cerebral ischemia and then once daily for 7 consecutive days, markedly ameliorated brain infarction and neurological deficits 7 days after reperfusion following 30 min of MCAo, whereas Non-acup-5Hz and Non-acup-25Hz did not reduce cerebral infarct areas or improve neurological status. Previous studies have reported that astrocytic activation contributes to increased infarct size through neuron-glial interactions during the subacute phase of cerebral ischemia, and that GFAP, a marker of reactive astrocytes, is predominantly expressed in the periinfarct border zone from 1 to 7 days after the onset of cerebral ischemia [30, 31]. Our western blot analyses revealed significantly upregulated GFAP expression in the ischemic cortical penumbra, and indicated that EA-5Hz and EA-25Hz both effectively downregulated GFAP expression in the ischemic cortical penumbra 7 days after reperfusion. Our and previous studies’ findings thus suggest that electroacupuncture stimulation at Baihui and Fengfu acupoints, but not at nonacupoints, at a frequency of 5 or 25Hz, exerts neuroprotective effects against cerebral I/R injury, and that such antiinfarct effects are, at least in part, caused by the downregulation of reactive astrocytosis-mediated neurotoxicity in the periinfarct cortex 7 days after reperfusion.

Previous studies have reported that MAPK and phosphatidylinositol-3-kniase (PI3K)/Akt signaling pathways are involved in the neuroprotective effects of EA against cerebral I/R injury in mild transient focal cerebral ischemia [32, 33]. The MAPK family consists of JNK, ERK1/2, and p38 MAPK proteins, which are activated by various stimuli, including growth hormones and cellular stresses [34]. Studies have shown that p38 MAPK signaling is involved in reactive astrogliosis and plays a crucial role in the synthesis of proinflammatory mediators in the cortical penumbra, leading to progressive infarction in the subacute phase of cerebral ischemia [1, 35]. Studies have also shown that active p38 MAPK expression in the periinfarct cortex or hippocampus peaks at 6 h, and lasts 1–3 days, after ischemia, and exerts neuroprotective effects against ischemic brain injury by activating antiapoptotic or CREB signaling pathways in ischemic [36], anesthetic [37] preconditioning, and global ischemia [38] models. A study done by Luo et al. has also demonstrated that the activation of Akt exerts neuroprotective actions against ischemic brain injury by activating the CREB signaling pathway in a neonatal rat model of cerebral hypoxia-ischemia [39]. CREB is a selective nuclear transcription factor that regulates the expression of genes for cell survival, neuroplasticity, and neurogenesis [40]. CREB phosphorylation (on Ser 133) predominantly occurs 1 h after ischemia and lasts up to 3 days followed by cAMP response element (CRE)-mediated gene transcription, which produces antiapoptotic proteins such as Bcl-2 and Bcl-xL in the periinfarct region after MCAo [41]. Some studies have demonstrated that HSP70 is a downstream target of p38 MAPK, associated with upregulated Bcl-2 and Bcl-xL expression in in vivo and in vitro models [42, 43]. Our immunoblot analysis results indicate that p-p38 MAPK was derived from total p38 MAPK, and that p-p38 MAPK, but not p-JNK, p-ERK1/2, or p-Akt, expression was markedly downregulated in the cytosolic fraction in the cortical penumbra; however, this expression was effectively restored by EA-5Hz and EA-25Hz 7 days after reperfusion. P-CREB, but not HSP70, immunoreactivity showed a similar pattern to cytosolic p-p38 MAPK immunoreactivity. Further analysis of p-CREB/DAPI costaining indicated p-CREB immunoreactivity in the cytoplasm and intense p-CREB immunoreactivity within the nuclear compartment in the cortical penumbra. These results are consistent with those of a previous study, which indicated that cytosolic p-CREB expression is positively associated with nuclear p-CREB expression and reflects the activation and nuclear translocation of CREB following hypoxic preconditioning [44]. Based on previous reports and our results, we propose that EA-5Hz and EA-25 Hz both provide neuroprotection against astrocyte-mediated toxicity, most likely by activating p38 MAPK signaling, and that their neuroprotective effects are possibly due to the activation of p38 MAPK/CREB, but not p38 MAPK/HSP70, signaling in the ischemic cortical penumbra 7 days after reperfusion.

Studies have shown that the protective effect of CREB phosphorylation against ischemia-induced neuronal death can be attributed to overexpression of the antiapoptotic proteins Bcl-2 and Bcl-xL [40, 45]. During cerebral ischemia, ischemic insults result in a reduced Bcl-2 (Bcl-xL)/Bax ratio and shift the balance toward apoptosis by promoting the release of cytochrome c, and Smac/DIABLO from mitochondria [45]. Pharmacological treatments increase the Bcl-2 (Bcl-xL)/Bax ratio, and shift the balance toward antiapoptotic effects by blocking the release of apoptogenic factors from mitochondria and subsequently inhibiting apoptotic cascades [45]. In previous studies, Bax expression was markedly upregulated, whereas Bcl-2 and Bcl-xL expression was significantly downregulated, in the ischemic region 1 day after transient cerebral ischemia [10, 46]. In this study, the expression of the proapoptotic protein Bax was predominantly upregulated, whereas that of the antiapoptotic protein Bcl-2 was significantly downregulated, in the cytosolic fraction of the ischemic cortical penumbra 7 days after reperfusion. Bax expression was markedly upregulated in the mitochondrial fraction, whereas Bcl-xL expression was downregulated. However, EA-5Hz effectively restored cytosolic and mitochondrial Bcl-xL expression, thereby contributing to the downregulation of mitochondrial Bax expression in the ischemic cortical penumbra. Interestingly, EA-25Hz prevented the downregulation of cytosolic Bcl-2 and mitochondrial Bcl-xL proteins, and significantly upregulated mitochondrial Bcl-2 expression, leading to abrogated cytosolic and mitochondrial Bax expression 7 days after reperfusion. EA-5Hz increased the Bcl-xL/Bax ratio in the mitochondrial fraction, which shifted the balance of the Bcl-2 family proteins toward survival. EA-25Hz markedly increased the Bcl-2/Bax and Bcl-xL/Bax ratios in the cytosolic fraction and strongly inhibited Bax translocation from the cytosol to the mitochondria, resulting in an increased mitochondrial Bcl-2/Bax ratio. This change exerted beneficial effects by stabilizing mitochondrial permeability transition after cerebral I/R injury. Studies have demonstrated that cytosolic p53, which is predominantly found in the ischemic region 12 h-2 days after ischemia, rapidly translocates to the mitochondria in response to cerebral ischemic insults, where it interacts with the pro- and antiapoptotic Bcl-2 family proteins and severely disrupts mitochondrial membrane integrity, leading to p53-mediated apoptosis [47, 48]. In this study, cytosolic and sparse mitochondrial (data not shown) p53 expression levels in the ischemic cortical penumbra were unaffected by EA-5Hz or EA-25Hz 7 days after reperfusion. Considering these and previous findings, we suggest that both EA-5Hz and EA-25Hz exert neuroprotective effects against Bax-mediated apoptosis, possibly due to the activation of p38 MAPK/CREB signaling, and that the downregulating effects of EA-5Hz and EA-25Hz on the insertion of Bax into mitochondria can be attributed to increased mitochondrial Bcl-xL/Bax and Bcl-2/Bax ratios, respectively, but not suppressed p53 signaling, in the cortical periinfarct area 7 days after reperfusion.

Evidence has suggested that Bax activation during apoptosis involves translocation to the mitochondria, insertion into the mitochondrial outer membrane, and formation of homo-oligomers, and that these processes lead to mitochondrial fragmentation and consequent release of apoptogenic proteins, including cytochrome c, Smac/DIABLO, and AIF [49–51]. The release of cytochrome c, coinciding with Bax translocation, facilitates the formation of the apoptosome, which binds and activates caspase-9 to initiate caspase-3-mediated apoptosis. This cascade is accompanied by Smac/DIABLO release to the cytosol, where Smac/DIABLO binds and neutralizes XIAP and prevents XIAP-mediated caspase suppression. By contrast, translocation of AIF from mitochondria to the nucleus initiates DNA fragmentation through a caspase-independent apoptotic pathway [52]. Druse et al. (2006) used an in vitro ethanol-treated cell culture model to show that the pharmacological restoration of Bcl-xL and XIAP expression plays a crucial role in neuroprotective effects against apoptosis [53]. Other research has found that activation of the extrinsic and intrinsic caspase-dependent apoptotic pathways, and activation of the caspase-independent apoptotic pathway occur in the penumbra zone, and shown the expression of cleaved caspase-8, cytochrome c, cleaved caspase-3, XIAP, and AIF in the cytosol after 4 h of reperfusion in a rat model of MCAo [52]. Our study results indicate that EA-5Hz and EA-25Hz did not affect the expression of cleaved caspase-8, and thus exerted no influence on extrinsic pathway-mediated (caspase-8-mediated) apoptosis. We observed upregulated cytosolic and mitochondrial Smac/DIABLO expression, upregulated cytosolic cleaved caspase-3 expression, and downregulated cytosolic XIAP expression, in the cortical penumbra after MCAo. However, EA-5Hz and EA-25Hz effectively reduced the extent of Smac/DIABLO and cleaved caspase-3 upregulation, and simultaneously restored XIAP expression, 7 days after reperfusion. Nonsignificant differences in the cytosolic and mitochondrial cytochrome c and AIF among the model and treatment groups indicated that EA-5Hz and EA-25Hz exert nonsignificant effects on cytochrome c- or AIF-mediated apoptosis. Our results thus strongly suggest that EA-5Hz and EA-25Hz are neuroprotective against Bax-mediated apoptosis by inhibiting the translocation of Smac/DIABLO from mitochondria to the cytosol, thereby restoring XIAP-mediated suppression of caspase-3 activity in the ischemic cortical penumbra 7 days after reperfusion. According to our research, this study is the first to show that EA at acupoints (5 and 25Hz) exerts neuroprotective effects by modulating Bcl-xL- and Bcl-2-mediated signaling pathways in the subacute phase after mild transient MCAo. Bcl-xL and Bcl-2 localize in different cell types and subcellular locations, and exert functionally different types of anti-apoptotic activity [54–56]. Bcl-xL has been shown to be more effective than Bcl-2 (approximately 10-fold) at inhibiting apoptosis in a cell culture model of breast cancer [56]. In this study, EA-25Hz-activated Bcl-2-mediated signaling influenced neurological recovery as early as 3 days after reperfusion, and the downregulating effects of Bcl-xL/Bax- and Bcl-2/Bax-mediated signaling on caspase-3 activation were similar 7 days after MCAo. However, further investigation is required to clarify the differences between these signaling pathways.

## Conclusions

Overall, our results suggest that EA-5Hz and EA-25Hz both effectively downregulate reactive astrocytosis to provide neuroprotection against cerebral infarction, most likely by activating p38 MAPK/CREB signaling. The modulating effects of EA-5Hz and EA-25Hz on Bax-mediated apoptosis are possibly due to the activation of p38 MAPK/CREB/Bcl-xL and p38 MAPK/CREB/Bcl-2 signaling, respectively, thereby preventing Smac/DIABLO translocation and restoring XIAP-mediated caspase-3 inhibition in the ischemic cortical penumbra 7 days after reperfusion. Our results indicate that electroacupuncture stimulation at the Baihui and Fengfu acupoints, at frequencies of 5 and 25Hz, provide promising therapeutic strategies in the subacute phase after MCAo. Further investigations are required to clarify the precise signaling mechanisms and the differences in these neuroprotective effects of EA-5Hz and EA-25Hz before future clinical application.

## Abbreviations

EA: Electroacupuncture
I/R: Ischemia-reperfusion
MAPK: Mitogen-activated protein kinases
JNK: C-Jun N-terminal kinase
ERK1/2: Extracellular signal-regulated kinase1/2
PI3K: Phosphatidylinositol-3-kniase
CREB: CAMP response element-binding protein
Smac/DIABLO: Second mitochondrial-derived activator of caspase /direct inhibitor of apoptosis -binding protein with low isoelectric point
XIAP: X-linked inhibitor of apoptosis protein
HSP70: Heat shock protein 70
GFAP: Glial fibrillary acidic protein
AIF: Apoptosis-inducing factor
COX IV: Cytochrome c oxidase subunit IV
DAPI: 4',6-diamidino-2-phenylindole
